# Mental health service use among mothers involved in public family law proceedings: linked data cohort study in South London 2007–2019

**DOI:** 10.1007/s00127-022-02221-1

**Published:** 2022-03-16

**Authors:** Rachel J. Pearson, Claire Grant, Linda Wijlaars, Emily Finch, Stuart Bedston, Karen Broadhurst, Ruth Gilbert

**Affiliations:** 1 Population, Policy and Practice Research and Teaching Department, UCL Great Ormond Street Institute of Child Health, London, WC1N 1EH UK; 2 Department of Epidemiology and Public Health, UCL Institute of Epidemiology and Health, London, WC1E 7HB UK; 3 Southwark and Addictions Directorate, South London and Maudsley NHS Foundation Trust, London, SE5 8RZ UK; 4 Centre for Child and Family Justice Research, Lancaster University, Lancaster, LA1 4YW UK

**Keywords:** Child protection, Maternal mental health, Family court, Record linkage, Substance misuse

## Abstract

**Purpose:**

Mental health problems and substance misuse are common among the mothers of children who experience court-mandated placement into care in England, yet there is limited research characterising these health needs to inform evidence-based policy. In this descriptive study, we aimed to generate evidence about the type, severity, and timing of mental health and substance misuse needs among women involved in public family law proceedings concerning child placement into care (‘care proceedings’).

**Methods:**

This is a retrospective, matched cohort study using linked family court and mental health service records for 2137 (66%) of the 3226 women involved in care proceedings between 2007 and 2019 in the South London and Maudsley NHS Mental Health Trust (SLaM) catchment area. We compared mental health service use and risk of dying with 17,096 female-matched controls who accessed SLaM between 2007 and 2019, aged 16–55 years, and were not involved in care proceedings.

**Results:**

Most women (79%) were known to SLaM before care proceedings began. Women had higher rates of schizophrenia spectrum disorders (19% vs 11% matched controls), personality disorders (21% vs 11%), and substance misuse (33% vs 12%). They were more likely to have a SLaM inpatient admission (27% vs 14%) or to be sectioned (19% vs 8%). Women had a 2.15 (95% CI 1.68–2.74) times greater hazard of dying, compared to matched controls, adjusted for age.

**Conclusion:**

Women involved in care proceedings experience a particularly high burden of severe and complex mental health and substance misuse need. Women’s increased risk of mortality following proceedings highlights that interventions responding to maternal mental health and substance misuse within family courts should offer continued, long-term support.

**Supplementary Information:**

The online version contains supplementary material available at 10.1007/s00127-022-02221-1.

## Introduction

There are high rates of mental health problems and substance misuse among mothers whose children are subject to public family law proceedings (‘care proceedings’) in England [1–4], prompting calls to strengthen interagency working between family courts, children’s social care and health services. Care proceedings are family court proceedings concerning whether a child (aged 0–17 years old) should be placed into state care to safeguard them from abuse or neglect. An estimated 75% of children in care proceedings are already in state care at onset of proceedings under interim legal orders or out-of-court arrangements and [4], at conclusion, around 50% of children are placed into care under a care order [5]. Other common legal orders made at the conclusion of proceedings include placement orders (i.e., placed to adopt) and special guardianship orders (typically used to place children with extended family). Around 2% of children receive no legal order. Earlier health support for birth parents may mitigate some of the child health risks associated with parental mental illness [6–10], including rare outcomes such as serious or fatal maltreatment [11]. It could also lead to fewer children requiring care proceedings, which are costly to the tax payer and one the most intrusive forms of child protection.

Since 2010, the number of applications made to initiate care proceedings (per 10,000 children) has risen by 400% in some parts of England, despite declining birth rates [5, 12]. There is currently limited evidence characterising parental mental health problems and mental health service use in relation to care proceedings [13–15]. Population-based characterisation of maternal mental health need and service use among birth mothers is needed, yet suitable data are lacking [16]. In several settings, researchers have overcome this barrier by linking administrative child protection and health datasets [17–21]. Within the SAIL Databank, Griffiths et al. used new linkages between Welsh family court data and health data on antenatal care, hospitalisations and general practitioner (GP) contacts, and found high prevalence of maternal mental health difficulties in the 2 years prior to childbirth [22]. The most common diagnoses were depression and anxiety [23]. In England, a dearth of similarly linked data is limiting the development of evidence-based policy for parental mental health and substance misuse in the context of child protection [16, 24].

In this descriptive study, we used a cohort of women involved in care proceedings who linked to de-identified patient records from a large London NHS mental health trust (serving ~ 1.4 million) [2, 25]. We characterised mental health service use between women in care proceedings who accessed mental health services and a matched control group comprising other women accessing mental health services to inform policy and service development across family justice, children’s social care, and health. The aim of this study was to describe the type, severity, and timing of mental health problems and substance misuse among women who linked, comparing them to a matched comparison group of other women using mental health services. To investigate long-term health outcomes, we looked at women’s risk of dying after care proceedings.

## Methods

This descriptive study has a retrospective, matched cohort design to enable comparison between our study cohort and a control population. This study follows the STROBE guidelines for reporting observational studies (Supplementary Table S1) [26].

### Data

We used longitudinal cohorts from the South London and Maudsley NHS Mental Health Trust (SLaM) case register [27]. SLaM provide secondary and tertiary mental health services, as well as the Improving Access to Psychological Therapies (IAPT) service which allows self-referral for common mental health disorders, to residents of the SLaM catchment area (Croydon, Lambeth, Lewisham and Southwark local authorities). De-identified SLaM patient records are stored in the Clinical Record Interactive Search (CRIS) database [28, 29], which has captured data from all SLaM services since January 2007 and from some services pre-2007. Due to poor accuracy of event dates before January 2005, we used an observation window of 1 January 2005 and 31 March 2020.

CRIS has been linked to Cafcass (Children and Family Court Advisory and Support Service) which contains information on all care proceedings in England since 1 April 2007 [2, 5]. The SLaM Clinical Data Linkage Service (CDLS) performed the linkage using a rule-based method with names, date of birth and postcode history, applying the separation principle. Under the separation principle, no one has access to a data set containing both person identifiers (e.g. names, date of birth and addresses) and attribute data (e.g. information about family court proceedings and mental health service use). This helps maintain the privacy of individuals in the linked data [30]. Of the 3226 women with a child involved in care proceedings in the SLaM catchment area between 1 April 2007 and 31 March 2019, 2137 (66.2%) linked to a SLaM patient record in November 2019 (when linkage occurred) [2].

We defined follow-up as the time (years) between a woman’s first contact with SLaM (referral, inpatient admission, or outpatient attendance) in the observation window and 31 March 2020, or death, whichever was earliest. Coverage of data used in this study is further described in Supplementary Fig. S1.

### Study population

Our study cohort included all women involved in care proceedings in the SLaM catchment area between 1 April 2007 and 31 March 2019, who linked to a SLaM patient record (*n* = 2137) [2]. Women in this cohort were typically younger at the birth of their first child than other women in England and most had just one child recorded in Cafcass. Half had an infant (< 12 months) subject to proceedings, consistent with prior research [31, 32]. A third had two or more sets of proceedings recorded in Cafcass between 2007 and 2019. Further information about this cohort and women who did not link can be found in our linkage report [2].

To understand how the study cohort differ from other women accessing mental health services, we constructed a comparator group (*n* = 153,486) comprising all women living in the SLaM catchment area who accessed SLaM services between 1 April 2007 and 31 March 2019, aged 16–55 years (i.e. reproductive age) (Supplementary Fig. S2). We excluded women who linked to Cafcass and had been involved in care proceedings between 2007 and 2019, either in the SLaM catchment area or in four neighbouring local authorities which SLaM provides some services to (Bexley, Bromley, Greenwich, Wandsworth). We exactly matched women in the comparator group to women in the study cohort based on the following strata: (1) having a record from a SLaM secondary and tertiary service, IAPT or both and (2) the calendar year of women’s first SLaM contact within the observation window. Women were grouped by matching strata and randomly selected without replacement at a ratio of 8:1, determined by the smallest matching strata, giving 17,096 matched controls. As SLaM do not routinely record information on pregnancy or births, we could not match on maternal status.

### Measures

#### Sociodemographic characteristics

Due to the poorer data availability in Cafcass for date of birth (4.2% missing) and ethnicity (44% missing) among our study cohort, we used these fields in CRIS where non-missing, and from Cafcass otherwise [2]. We used women’s date of birth to derive age at first SLaM contact. Women’s ethnicity was presented in the following five categories based on the NHS 16 + 1 ethnic data categories: Asian or Asian British, Black or Black British, Mixed heritage, White, Other ethnic background [33].

#### Severity and intensity of mental health service use

We categorised SLaM service use within the observation window into four types of activity: (1) referrals (accepted or rejected by the service), (2) outpatient appointments (planned and attended), (3) inpatient admissions and (4) being sectioned under the mental health act (i.e., being detained in hospital for assessment or treatment). We further categorised referrals and outpatient appointments by whether the SLaM service was IAPT. We also derived measures indicative of women’s engagement with services. This included discharged referrals due to ‘failure to engage’ (i.e. persistent non-attendance or poor engagement with the service) and the proportion of outpatient appointments over the observation window that were missed due to non-attendance, attending too late to be seen, or patient cancellation. For study cohort women, we calculated the time between their first SLaM contact over the observation window and their first recorded (‘index’) set of care proceedings in Cafcass.

#### Mental health and behavioural diagnoses

Psychiatric diagnoses were captured in structured fields recorded in CRIS using ICD-10 codes [34]. Diagnoses were also extracted from free-text fields using natural language processing applications developed by the NIHR Maudsley Biomedical Research Centre [28]. As we did not require exact dates for diagnoses, we included diagnoses made before 1 January 2005. We grouped mental and behavioural disorder diagnoses (ICD-10 Chapter V) into seven categories and defined serious mental illness (Table 1).

**Table 1:** ICD-10 codes used to identify mental health and behavioural disorder diagnoses in CRIS records

Diagnosis categories	ICD-10 codes
Schizophrenia, schizotypal and delusional disorders	F20-29
Severe mood disorders (i.e. bipolar affective disorder, severe or moderate depressive disorders, puerperal psychosis and postnatal depression)	F30-31, F32.1-32.3, F33.1-33.3, F34.0-34.1, F53.0-53.1
Anxiety, somatoform and stress-related disorders	F40-48
Other depressive disorders	F32.0, F32.8-32.9, F33.0, F33.4-33.9, F34.8-34.9, F38-39
Drug and alcohol-related disorders	F10-19 (excluding F17)
Personality disorders	F60-63
Other psychiatric disorders (including eating disorders, other perinatal psychiatric disorders and ‘Unspecified mental illness’)	F50-3, F53.8-53.9, F99
Disorders of psychological development and behavioural and emotional disorders with onset usually occurring in childhood or adolescence	F80-F89, F90-F98

We defined serious mental illness as severe mood disorders or schizophrenia, schizotypal and delusional disorders

*ICD-10* International Statistical Classification of Diseases and Related Health Problems 10th Revision

#### Substance misuse and learning disabilities

Women were coded as having a record of substance misuse if they had a substance misuse-related diagnosis [35], or accessed any SLaM substance misuse services (excluding services for smoking). To investigate dual-diagnosis (both psychiatric and substance misuse diagnoses), we identified women with both a record of substance misuse and a psychiatric diagnosis (Table 1), excluding drug and alcohol-related psychiatric disorders. Women were coded as having a learning disability if they had a related ICD-10 diagnosis [36], or accessed any SLaM services for people with learning disabilities.

#### Specialist mental health service use

We explored use of several specialist mental health services including: perinatal (including a mother-baby unit); psychosis; acute (e.g. places of safety, home treatment teams, and short- and long-term inpatient care); forensic, criminal justice-related (e.g. non-forensic services providing psychiatric care to individuals involved in criminal justice system or as part of a criminal sentence); substance misuse; child and adolescent mental health services (CAMHS); and parent or whole family (e.g. CAMHS family services, parenting assessment or support, and parent–child interaction services). Service groups were not mutually exclusive as some services fit into two or more groups.

#### Date of death

We used the ‘date of death’ field in CRIS to identify deaths within the observation window and to derive age at death [28].

### Statistical analysis

We described study measures using frequencies and percentages or medians with 25th and 75th percentiles, stratified by group (study cohort or matched controls). To account for age differences between groups, we age-standardised percentages for the matched controls, with the study cohort as the reference population (age categories: 0–17 years old, 18–24, 25–29, 30–34, 35–39, 40–44, 45–49, 50 +).

#### Mortality and immortal time bias

To compare mortality between the two groups, we had to first account for immortal time bias among women involved in care proceedings. Women in the care proceedings cohort whose index care proceedings began after their first SLaM contact had to be alive at least until their index proceedings began, whereas there was no such restriction among the matched controls. If we had not accounted for this bias, the difference in mortality over follow-up between study cohort and the matched controls may have been underestimated. To correct for this bias, we used Cox proportional hazards models with death over follow-up (died = 1, alive/censored = 0) as our model outcome and involvement in care proceedings (yes = 1, no = 0) and age at first SLaM contact (years) as model covariates, with involvement in care proceedings included as a time-varying covariate. This meant that, for women whose index proceedings began after their first SLaM contact, we split their follow-up time in two at the start date of index proceedings. The time before they entered care proceedings was coded as 0 to reflect that they were unexposed during this period (e.g. immortal person-time) and the time from the start of their index proceedings to the end of follow-up was coded as 1 (reflecting the period after onset of care proceedings). Three of the matched controls (0.5% of matched controls that died and 0.02% of all matched controls) were excluded from modelling as their date of death occurred before their first SLaM contact. We visually assessed the proportional hazards assumption using Schoenfeld residual plots and also tested the correlation between the Schoenfeld residuals and survival time using the cox.zph function in the R survival package [37]. We fitted alternative models allowing the independent association between age at first SLaM contact and death to be non-linear using quadratic and, later, natural spline functions (Supplementary Figs. S3 and S4). We checked for influential observations using dfbeta value plots and found no evidence of outliers (Supplementary Fig. S5). As a sensitivity analysis, we report findings from the model without accounting for immortal time bias (Supplementary Table S2).

Using our model, we calculated expected mortality rates at 5 and 10 years from first SLaM contact for the study cohort and the matched controls via the Aalen–Johansen method. For these predictions, we held age constant at the 25%, 50% and 75% quantiles of age at first SLaM contact among the study cohort. We derived asymptotic 95% confidence intervals for these rates using the log–log transformation. We used the survival package in R [37].

### Ethical approval and patient and public engagement

This study forms part of a wider project linking routinely collected pseudonymised data on care proceedings and health without individual patient consent [38]. We received approval from an NHS Research Ethics Committee (ref: 18/SC/0363), the NHS Confidentiality Advisory Group (ref: 18/CAG/0112), the CRIS Oversight Committee (ref: 19-050), and the Cafcass Research Governance Committee. We carried out several public engagement activities to gain feedback on the acceptability of linking these data for research, as described previously [2].

## Results

Most study cohort women (*n* = 1686, 78.9%) were known to SLaM before their index set of care proceedings began (median time from first SLaM contact to index proceedings: 2.3 years; 25% quantile: 5.5 years, 75% quantile: 53 days).

### Matching variables

There was exact balance among the matching variables (Supplementary Table S3). Most study cohort women (*n* = 1948, 91.2%) were referred to, or accessed, SLaM secondary or tertiary mental health services within the observation window, half accessed specialist mental health services and IAPT services (*n* = 922, 43.1%), and few accessed IAPT services alone (*n* = 189, 8.8%). The median length of follow-up from first SLaM contact was 10.6 years (Table 2).

**Table 2:** Severity and intensity of SLaM service use over follow-up among the study cohort (*n* = 2137) and the matched controls (*n* = 17,096)

Characteristics of service use over the observation window (1 January 2005 to 31 March 2020)	Frequency (%)^a^ or Median [25%, 75% quantile] among women using SLaM services	
	Study cohort (*n* = 2137)	Matched controls (*n* = 17,096)
Follow-up time		
Time from first SLaM contact to 31st March 2020 or death	10.63 [7.01, 13.19]	10.59 [6.99, 13.16]
IAPT		
Any accepted referrals	1012 (47.4)	9210 (55.2)
Median number of accepted referrals per woman	2.00 [1.00, 3.00]	2.00 [1.00, 3.00]
Any rejected referrals	301 (14.1)	1612 ( 9.6)
Ever discharged from an active referral due to failure to engage (among those ever accepted to IAPT)	767 (75.8)	5691 (61.8)
Any IAPT attendance	748 (35.0)	7598 (45.4)
Median proportion of planned IAPT attendances that were missed or cancelled (per woman)	0.33 [0.00, 0.50]	0.22 [0.00, 0.42]
Secondary or tertiary mental health services		
Any accepted referrals	1817 (85.0)	14,287 (83.7)
Median number of accepted referrals per woman	3.00 [1.00, 5.00]	2.00 [1.00, 3.00]
Any rejected referrals	726 (34.0)	4130 (24.5)
Ever discharged from an active referral due to failure to engage (among those ever accepted to secondary/tertiary services)	712 (39.2)	2874 (20.1)
Any outpatient attendances	1847 (86.4)	14,882 (86.7)
Median proportion of planned outpatient attendances that were missed or cancelled (per woman)	0.17 [0.09, 0.28]	0.11 [0.00, 0.25]
Any SLaM inpatient admissions	582 (27.2)	2373 (13.4)
Median number of admissions per woman	2.00 [1.00, 4.00]	1.00 [1.00, 3.00]
Median average length of inpatient stay (days) per woman	27.00 [11.30, 52.30]	23.00 [8.00, 52.00]
Ever sectioned under the Mental Health Act 1983	404 (18.9)	1287 (7.2)
Section 135 or 136 (i.e. police)	226 (10.6)	530 (3.1)
Section 2 or 3 (i.e. assessment or treatment)	331 (15.5)	1081 (6.0)
Sections related to criminal justice^b^	15 (0.7)	34 (0.2)

Medians are calculated from the subset of women who have ever had the qualifying event

*IAPT* improving access to psychological therapies; *SLaM* South London and Maudsley

^a^Age-standardised rates are presented in brackets ‘()’ for the matched controls, with the study cohort as the reference population

^b^Sections 35–37, 41, 46–48 of the Mental Health Act 1983

### How women involved in care proceedings differ from other women accessing SLaM services

#### Age and ethnicity

All women had their date of birth recorded in CRIS. Study cohort women were typically younger at their first SLaM contact (median age: 28.2 years), compared to matched controls (30.7 years). Half of women were from a White ethnic background (study cohort: 48.9%; matched controls: 52.7%). More study cohort women were from Black (32.8% vs 22.1%) or mixed (7.0% vs 4.8%) ethnic backgrounds, and fewer had from Asian ethnic backgrounds (2.1% vs 2.7%), with unknown ethnicity (2.6% vs 7.2%) or Other ethnic backgrounds category (6.7% vs 10.6%), compared to matched controls. Age and ethnicity are further described in Supplementary Table S4.

#### Severity and intensity of mental health service use

Half of women had an accepted IAPT referral, though more study cohort women had a rejected IAPT referral (Table 2). Compared to matched controls, fewer study cohort women had an IAPT attendance. Among women with a planned IAPT attendance, the study cohort typically missed a greater proportion of planned attendances, compared to the matched controls. Most women with an accepted IAPT referral were discharged due to failure to engage, though this was more prevalent among the study cohort.

Over 80% of women had an accepted referral to secondary or tertiary SLaM services. Again, more study cohort women had a rejected referral. Over 85% of women had an outpatient attendance, though the study cohort typically missed a greater proportion of planned attendances compared to the matched controls. Twice as many study cohort women with an accepted referral were discharged due to failure to engage, compared to matched controls.

Compared to matched controls, study cohort women were twice as likely to have a SLaM inpatient admission, and of those admitted, were more likely to have a greater number of admissions and longer inpatient stays. Almost one-fifth of study cohort women were sectioned under the Mental Health Act 1983 and three times as many were sectioned by police compared to matched controls.

#### Mental health and behavioural diagnoses

Most study cohort women (*n* = 1747, 81.8%) and the matched controls (13,180, 76.8%) had at least one mental health or behavioural diagnosis among the Table 1 categories. More study cohort women (*n* = 1096, 51.3%) than matched controls (*n* = 6599, 38.5%) had diagnoses in two or more categories.

**Fig. 1:**
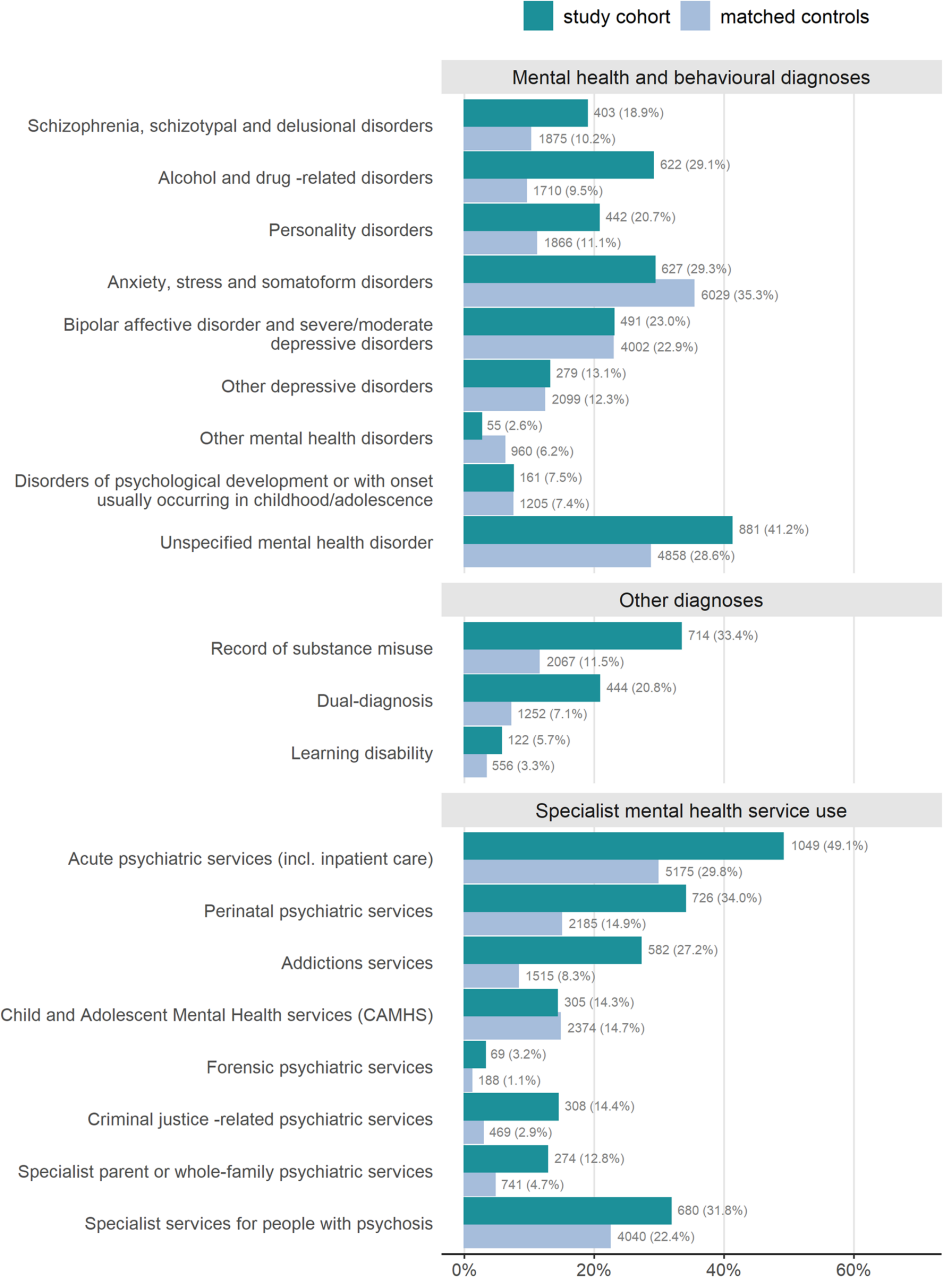
Prevalence (%) of mental health and behavioural diagnoses, other diagnoses, and specialist mental health service use among the care proceedings cohort (*n* = 2137) and the matched controls (*n* = 17,096). Age-standardised percentages are given for the matched controls.]

Prevalence of anxiety, stress and somatoform disorders, severe mood disorders, and other depressive disorders was similar between study groups (Fig. 1). Compared to matched controls, twice as many study cohort women had diagnoses of schizophrenia, schizotypal and delusional disorders or personality disorders. We presented ‘unspecified mental health disorder’ separately from the ‘other mental health disorders’ category due to its high prevalence. Among women with an ‘unspecified mental health disorder’ diagnosis, most had diagnoses in other Table 1 categories (study cohort: *n* = 708, 80.4%; matched controls: *n* = 3594, 74.0%).

Alcohol or drug-related diagnoses were more prevalent among the study cohort, compared to matched controls. Differences between the two groups were greater for drug-related diagnosis than for alcohol-related diagnosis (Supplementary Table S5). Slightly more study cohort women (*n* = 705, 33.0%) had a serious mental illness compared to matched controls (*n* = 5124, 28.9%). Among study cohort women, most diagnoses were recorded before their index set of care proceedings (Supplementary Fig. S6).

#### Substance misuse and learning disabilities

A third of study cohort women had a record of substance misuse, twice as many as in the matched controls (Fig. 1). A fifth of study cohort women had both a record of substance misuse and a non- drug- or alcohol-related psychiatric diagnosis, three times as many as in the matched controls. Few women had a learning disability.

Further investigation into multiple diagnoses found that one in ten study cohort women had a serious mental illness diagnosis, substance misuse, and at least one other mental health disorder diagnosis, over their life course (Supplementary Fig. S7).

#### Specialist mental health service use

Half of study cohort women accessed acute psychiatric services compared with under a third of the matched controls (Fig. 1). Study cohort women were more likely to have accessed SLaM psychosis, forensic, or substance misuse services, and four times as likely to have accessed SLaM criminal justice-related services, compared to matched controls. One-third of study cohort women accessed SLaM perinatal services. Few study cohort women (*n* = 95, 4.4%) accessed the SLaM mother-baby unit.

#### Mortality up to 31 March 2020

**Fig. 2:**
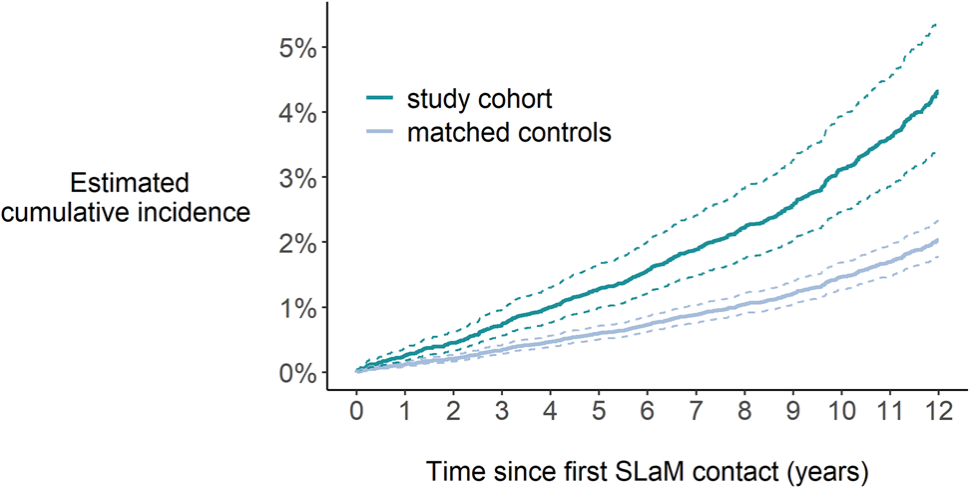
Estimated cumulative incidence of dying among women, who were aged 28 years old at their first SLaM contact in both the women in care proceedings cohort (*n* = 2137) and the matched controls (*n* = 17,096). *SLaM* South London and Maudsley.]

Seventy-seven study cohort women (3.6%) and 587 matched controls (2.6%, age-standardised) died within the observation window. Among study cohort women, 75% of women who died were under 48 years old. After performing model checks, the final model included age as a linear effect on death. Accounting for immortal time bias and adjusting for age at first SLaM contact, study cohort women had a 2.15 (95% CI 1.68–2.74) times greater hazard of dying than the matched controls (Supplementary Table S2). From our model, study cohort women and aged 28 years old at their first SLaM contact had an expected 5-year mortality rate of 1.29% (95% CI 0.99–1.66%), compared to 0.60% (95% CI 0.51–0.71%) for women in the matched controls aged 28 years old (Fig. 2); the expected 10-year mortality rate was 3.12% (95% CI 2.47–3.94%), compared to 1.46% (95% CI 1.27–1.68%) among the matched controls. Further estimated mortality rates are included in Supplementary Table S6.

## Discussion

### Summary of main findings

In this study, we described mental health service use among women involved in care proceedings in the SLaM catchment area. Of the 66.2% of women with a SLaM patient record, most were known to SLaM prior to proceedings commencing, indicating high prevalence of pre-existing or concurrent mental health need. Overall, 54.2% of the 3226 women involved in care proceedings in the SLaM catchment area between 2007 and 2019 had a formal mental health diagnosis recorded by SLaM.

Women in the study cohort had higher rates of inpatient admissions, contacts with acute, psychosis, and criminal justice-related psychiatric services and being sectioned under the Mental Health Act 1983 compared to a matched control group. They also demonstrated a higher burden of serious and complex mental illness, with twice as many women diagnosed with schizophrenia spectrum disorders, personality disorders, and substance misuse compared to the matched controls. After adjusting for age, study cohort women had higher mortality rates than the matched control group.

### Findings in context

Our findings complement a small, but growing body of research identifying high maternal mental health need among women involved in care proceedings using routinely collected records [1, 3, 23, 39]. We found higher prevalence of mental health need among women involved in care proceedings than previously reported in Wales [22]. However, this may be explained by our inclusion of all mothers involved in care proceedings, with Griffith’s et al. describing mothers of infants ‘born into care’. We also used linkage to SLaM record to identify mental health need, rather than relying on clinical diagnoses made during hospitalisation or GP contact, and were able to follow women for over 12 years. In Wales, 40% of women with an infant in care proceedings had a depression diagnosis recorded in the 2 years before birth [23]. In this study, we split mood disorders into ‘severe mood disorders’ and ‘other depressive disorders’ making comparison difficult. Rates of serious mental illness in the Welsh study (defined as schizophrenia spectrum disorders and bipolar affective disorders) were low (4%). However, 403 women (12% of 3226) in our care proceedings cohort had a diagnosis of schizophrenia spectrum disorder at some stage. This suggests prevalence of serious mental illness among women involved in care proceedings is higher in South London than in Wales. We also found higher rates of anxiety in South London (627 out of 3226; 19% vs 11% in Wales), which may partly be explained by the impact of proceedings on women’s mental health as we captured diagnoses recorded before, during and after proceedings [23, 40]. Finally, we found that women involved in proceedings had higher mortality rates compared with matched controls, adjusting for age. Qualitative evidence about the experiences of women in England following care proceedings describes the ‘collateral consequences’ of having children placed into care on mothers’ material and social support [40, 41]. For example, women living in social housing or in receipt of benefits may become ineligible for some welfare payments or become subject to the bedroom tax or even be moved to a smaller council-owned home. [41] Women also often report experiences of social stigmatisation and judgement from friends, family and communities, which compound their own feelings of failure and of loss of their identity as a parent [40]. Women who experience mental health problems and substance misuse problems, are likely to find it difficult to cope with these cumulative stressors. In this study, we did not attempt adjustment for other risk factors for premature death as we were limited to answering how mortality rates differed between our study cohort and matched controls rather than why they differed. Previous research into deaths among women whose children entered care in Manitoba, Canada and Sweden found that the association between entry into care and death remains after adjusting for other mortality risk factors [17, 42, 43]. These studies also used several control group methods including biological sisters and women whose children died. Future linkages in England with other health datasets, including Hospital Episodes Statistics (HES), will be essential to understanding the life-long health needs of women involved in care proceedings.

### Strengths and limitations

We have addressed gaps in the evidence base to provide comprehensive description of mental health service use among women involved in care proceedings in England. Though the SLaM population may vary in terms of population demographics and service availability from other parts of the UK—potentially affecting the generalisability of these findings—we found similarly high rates of mental health need among women involved in proceedings as were found in Wales [22]. Another key strength of this study is the use of linked administrative data between public family law and mental health records [2]. Analysis of routinely collected data offers unique insight into individual-level health service patterns for vulnerable mothers and mitigates common challenges with longitudinal research, such as selection bias, self-reporting bias and attrition [5, 28]. However, as data in Cafcass and CRIS are primarily collected for administrative purposes and not research, this study is limited by the scope and quality of available data.

First, it is unclear whether having no diagnosis in CRIS represents no mental health service need, lack of contact with services, or poor recording. It is also possible that clinicians are more reluctant to diagnose patients with complex or stigmatising conditions, such as personality disorders [44]. Similarly, we were unable to identify missingness among service use measures.

Second, this study is limited in understanding barriers to SLaM clinical care, such as reasons for rejected referrals and patient disengagement. For example, we could not calculate individual waiting times between referral, assessment, and treatment in these data.

Third, data on wider health services, including GPs and non-SLaM hospitals, were not available to us. For example, women with mental health problems that were adequately treated by GPs would not be represented in this study. Furthermore, changes in substance misuse service provision to non-SLaM providers within the SLaM catchment area over the observation window mean that the prevalence of substance misuse is likely underestimated. Mental health records do not routinely capture physical health issues, or characteristics that impact on health, including domestic violence, and are limited in their ability to investigate mortality, with no cause of death available. We also have no information about migration out of the SLaM catchment area which could lead to an underestimation of diagnoses and SLaM activity in either group.

Fourth, we were unable to match controls on maternal status. Hospital maternity indicators would allow further investigation into differences in characteristics of SLaM service use, diagnoses, and mortality, which may be partly explained by motherhood. Nevertheless, recent research from Wales, where researchers were able to construct a matched control group of mothers for women in care proceedings with infants, found similar evidence of higher rates of mental health disorders and substance misuse before birth among women in care proceedings [22].

Finally, women’s index set of care proceedings may not have been their first set of care proceedings as we only had data on proceedings between April 2007 and March 2019. Similarly, the CRIS data are only complete from January 2007; therefore, where women had SLaM service use before 2007, this may not have been captured. However, 78.9% of women whose index set of proceedings began between April 2008 and March 2019 had a prior SLaM contact.

### Implications of the findings

Our findings support recent work from Wales in making the case for integrating adult mental health services within children’s social care and family court practice. In both settings, large numbers of women with children in care proceedings demonstrated pre-existing mental health or substance misuse needs that are wide ranging and often complex [22, 23]. Timely access to mental health services for women at risk of care proceedings would likely improve outcomes for women and their families, therefore, more effort should be made to accelerate access for parents, particularly those in care proceedings. Previous research highlights the often multiple barriers women face in accessing mental health services during child protection procedures, such as care proceedings [45]. For example, specialist perinatal services can be small and oversubscribed [46], and often ineligible to women whose children are in out-of-home care. Understanding and addressing these barriers to support more women to have meaningful engagement with mental health services should be a key priority for policy-makers and commissioners, especially around pregnancy and childbirth [2].

We show that services targeted to women involved in care proceedings must be equipped to respond to complex and acute mental health need, as well as common mental health disorders and substance misuse. In recognising multiple needs, services must be multi-disciplinary or have strong referral pathways across health, social care, and voluntary organisations. For example, current commissioning of substance misuse services in South London from non-SLAM providers has contributed to poorly integrated care models within SLaM, and more can be done to optimise collaboration between services [47].

## Supplementary Information

Below is the link to the electronic supplementary material.

Supplementary file1 (DOCX 1676 KB)

## Data Availability

The data that support the findings of this study are not publicly available and require approval from an NHS Research Ethics Committee and the Clinical Record Interactive Search (CRIS) Oversight Committee. Use of these linked data is encouraged and interested researchers should contact the CRIS administrator at cris.administrator@slam.nhs.uk.

## Code availability

Data extraction was performed in SQL and data analysis was performed in R. Code is available on request from rachel.pearson@ucl.ac.uk.
