# Supplementary material for: Mental health service use among mothers involved in public family law proceedings: linked data cohort study in South London 2007–2019
Source: Soc Psychiatry Psychiatr Epidemiol. 2022 Mar 16;57(10):2097–108. doi: 10.1007/s00127-022-02221-1 (PMC9477900; doi:10.1007/s00127-022-02221-1)
Supplement: Supplementary file 1 — Supplementary file1 (DOCX 1676 KB) [file 127_2022_2221_MOESM1_ESM.docx]

Mental health service use among mothers involved in public family law proceedings: a linked data cohort study. Appendix

**Figures:**

[Figure S 1: Coverage of the data used in this study 2](file:///S:\ICH_PPP_CENB_CEBCH\Legal%20Epidemiology\Nuffield%20Cafcass-CRIS-HES%20project\Outputs\Working%20papers\CRIS-Cafcass%20papers\Descriptive%20paper\Appendix%20-%20updated%20-%20clean.docx#_Toc80017292)

[Figure S 2: Identifying a comparator cohort. Women aged 16-55 years old, accessing SLaM secondary and tertiary services and IAPT between April 2007 and March 2019, with an address history in the SLaM catchment area (Croydon, Lambeth, Lewisham or Southwark) 3](file:///S:\ICH_PPP_CENB_CEBCH\Legal%20Epidemiology\Nuffield%20Cafcass-CRIS-HES%20project\Outputs\Working%20papers\CRIS-Cafcass%20papers\Descriptive%20paper\Appendix%20-%20updated%20-%20clean.docx#_Toc80017293)

[Figure S 3: Schoenfeld residuals plot for model covariates (membership of the care proceedings cohort – top; age at first SLaM contact – bottom) in the cox model investigating mortality 4](file:///S:\ICH_PPP_CENB_CEBCH\Legal%20Epidemiology\Nuffield%20Cafcass-CRIS-HES%20project\Outputs\Working%20papers\CRIS-Cafcass%20papers\Descriptive%20paper\Appendix%20-%20updated%20-%20clean.docx#_Toc80017294)

[Figure S 4: Checking for non-linear effect of age at first SLaM contact on mortality 4](file:///S:\ICH_PPP_CENB_CEBCH\Legal%20Epidemiology\Nuffield%20Cafcass-CRIS-HES%20project\Outputs\Working%20papers\CRIS-Cafcass%20papers\Descriptive%20paper\Appendix%20-%20updated%20-%20clean.docx#_Toc80017295)

[Figure S 5: dfbeta values plot for model covariates (age at first SLaM contact – left) ; membership of the care proceedings cohort – right) in the cox model investigating mortality 5](file:///S:\ICH_PPP_CENB_CEBCH\Legal%20Epidemiology\Nuffield%20Cafcass-CRIS-HES%20project\Outputs\Working%20papers\CRIS-Cafcass%20papers\Descriptive%20paper\Appendix%20-%20updated%20-%20clean.docx#_Toc80017296)

[Figure S 6: Timing of diagnoses relative to women’s index set of care proceedings (n = 1747 out of 2137, 82%), by diagnosis type 6](file:///S:\ICH_PPP_CENB_CEBCH\Legal%20Epidemiology\Nuffield%20Cafcass-CRIS-HES%20project\Outputs\Working%20papers\CRIS-Cafcass%20papers\Descriptive%20paper\Appendix%20-%20updated%20-%20clean.docx#_Toc80017297)

[Figure S 7: Multiple diagnoses among women involved in care proceedings and known to SLaM services (n = 2137) 7](file:///S:\ICH_PPP_CENB_CEBCH\Legal%20Epidemiology\Nuffield%20Cafcass-CRIS-HES%20project\Outputs\Working%20papers\CRIS-Cafcass%20papers\Descriptive%20paper\Appendix%20-%20updated%20-%20clean.docx#_Toc80017298)

**Tables:**

[Table S 1: STROBE checklist 8](#_Toc88904998)

[Table S 2: Cox proportional hazards model results (top two rows – final reported model; bottom two rows – final model but without ‘Involvement in care proceedings’ as a time-dependent covariate) 10](#_Toc88904999)

[Table S 3: Distribution of the matching variable, by cohort 11](#_Toc88905000)

[Table S 4: Age and ethnicity, by cohort 12](#_Toc88905001)

[Table S 5: Substance-related psychiatric diagnoses, by cohort 12](#_Toc88905002)

[Table S 6: Estimated 5 and 10-year mortality rates for women aged 21, 28, and 36 years old at first SLaM contact 13](#_Toc88905003)

Figure S 1: Coverage of the data used in this study


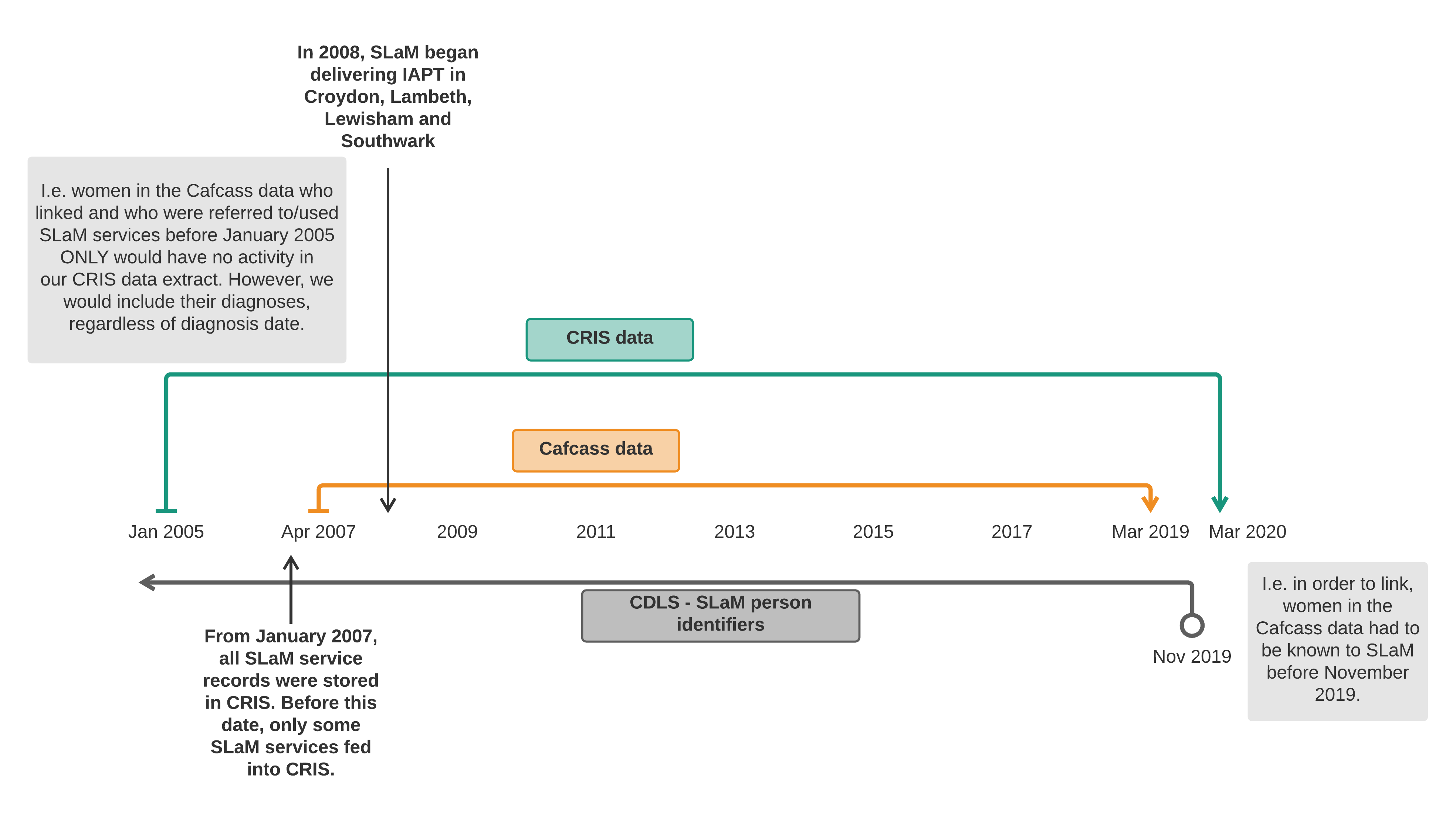

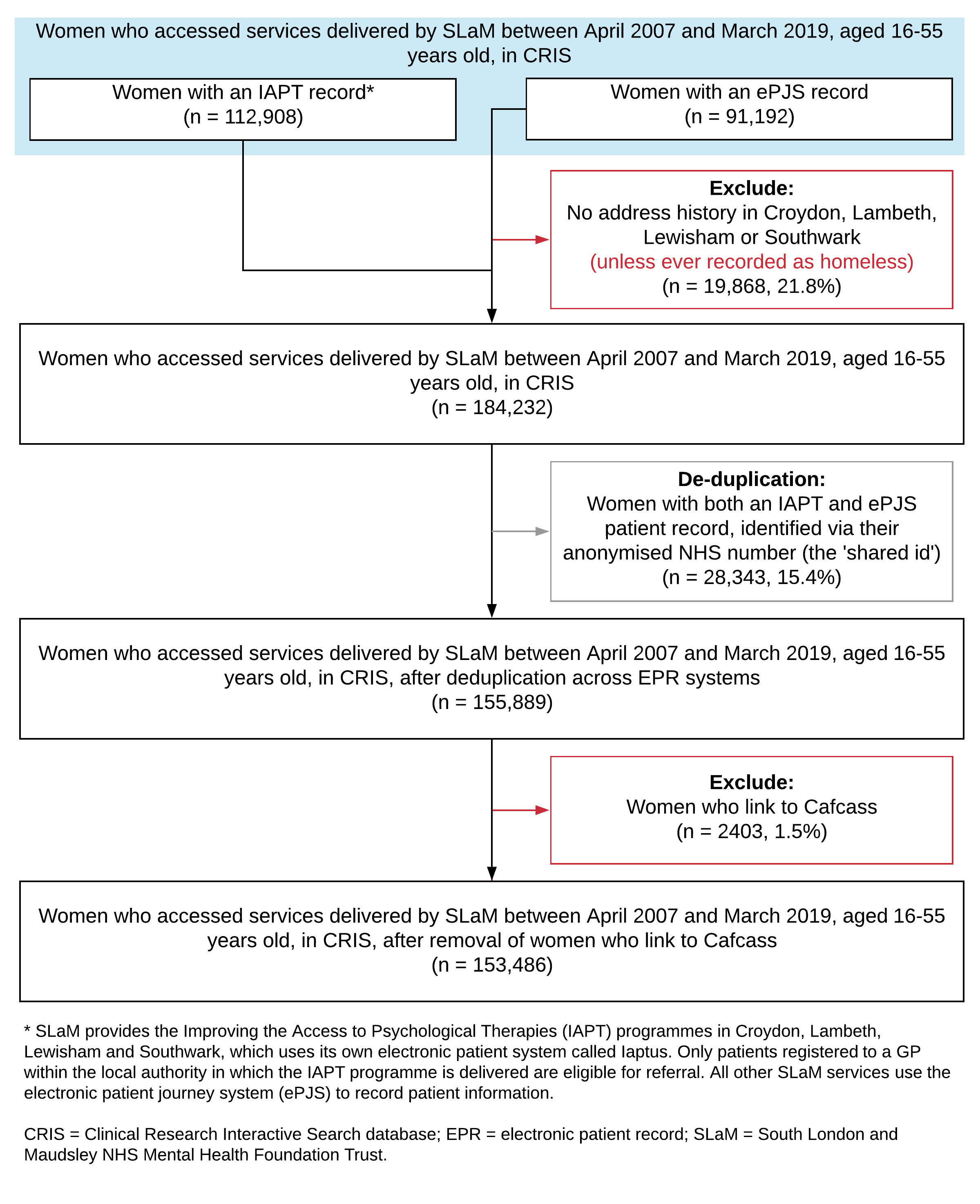


Figure S 2: Identifying a comparator cohort. Women aged 16-55 years old, accessing SLaM secondary and tertiary services and IAPT between April 2007 and March 2019, with an address history in the SLaM catchment area (Croydon, Lambeth, Lewisham or Southwark)


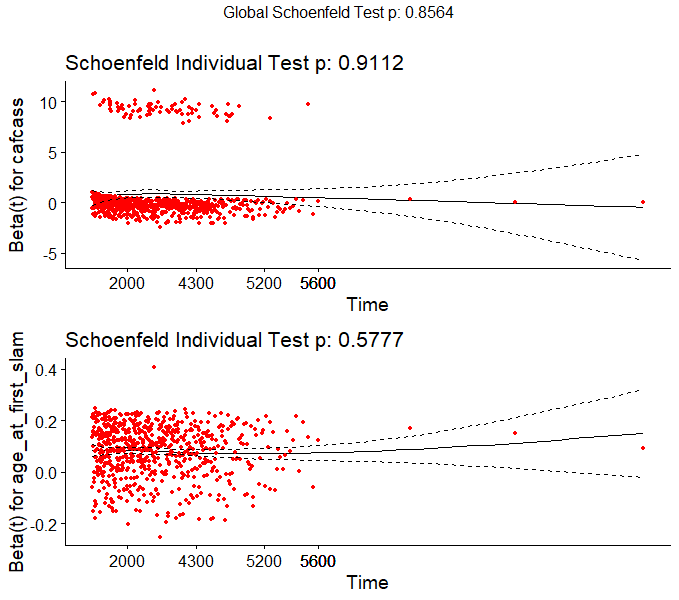


Figure S 3: Schoenfeld residuals plot for model covariates (membership of the care proceedings cohort – top; age at first SLaM contact – bottom) in the cox model investigating mortality

Likelihood ratio tests between the model with age as a quadratic effect and age as a linear effect and between the model with age modelled using a natural cubic spline and age as a linear effect yielded no evidence of a non-linear relationship between death over follow-up and age at first SLaM contact.


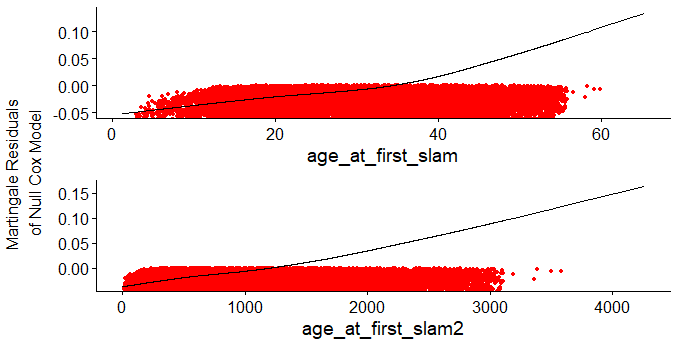


Figure S 4: Checking for non-linear effect of age at first SLaM contact on mortality


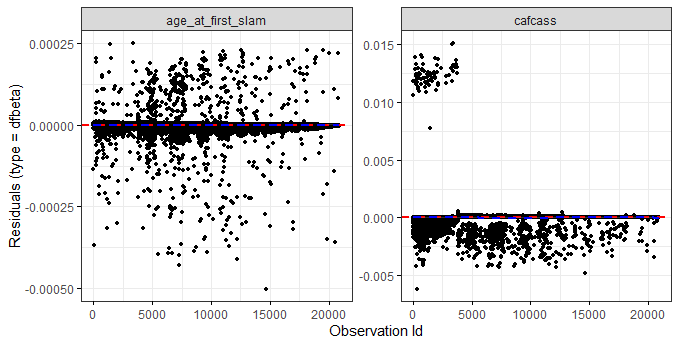


Figure S 5: dfbeta values plot for model covariates (age at first SLaM contact – left) ; membership of the care proceedings cohort – right) in the cox model investigating mortality


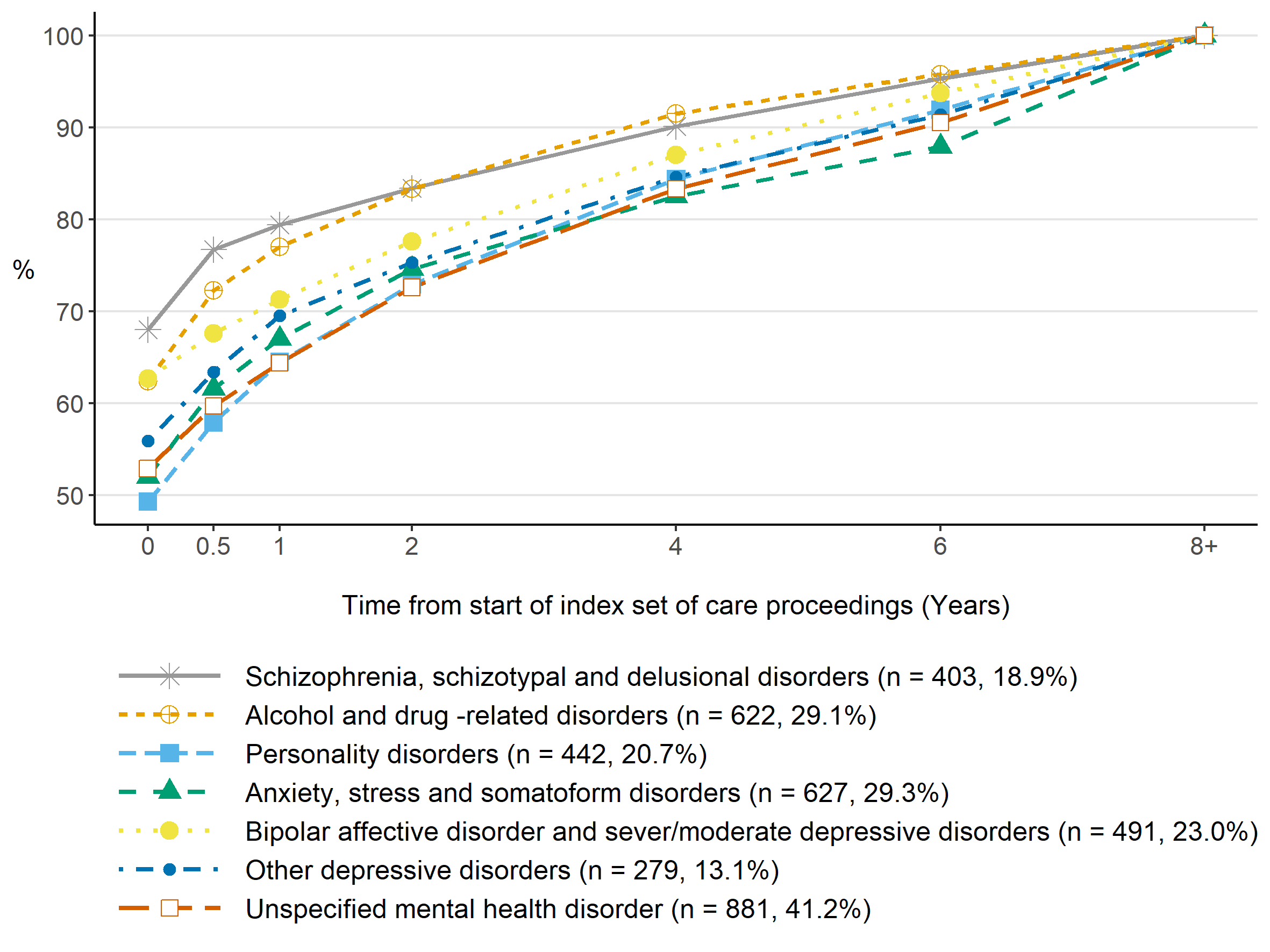


Figure S 6: Timing of diagnoses relative to women’s index set of care proceedings (n = 1747 out of 2137, 82%), by diagnosis type

Note: the percentages plotted at time 0 refer to the proportion of women with that diagnosis in the SLaM data who received the diagnosis before their index (first recorded) set of care proceedings began. The percentages in the legend refer to the percentage of women in the study cohort (n = 2137) who have that diagnosis in the SLaM data. The ‘Other mental health disorders’ and ‘Disorders of psychological development or with onset usually occurring in childhood/adolescence’ categories could not be shown due to small cell counts (< 10)

Figure S 7: Multiple diagnoses among women involved in care proceedings and known to SLaM services (n = 2137)


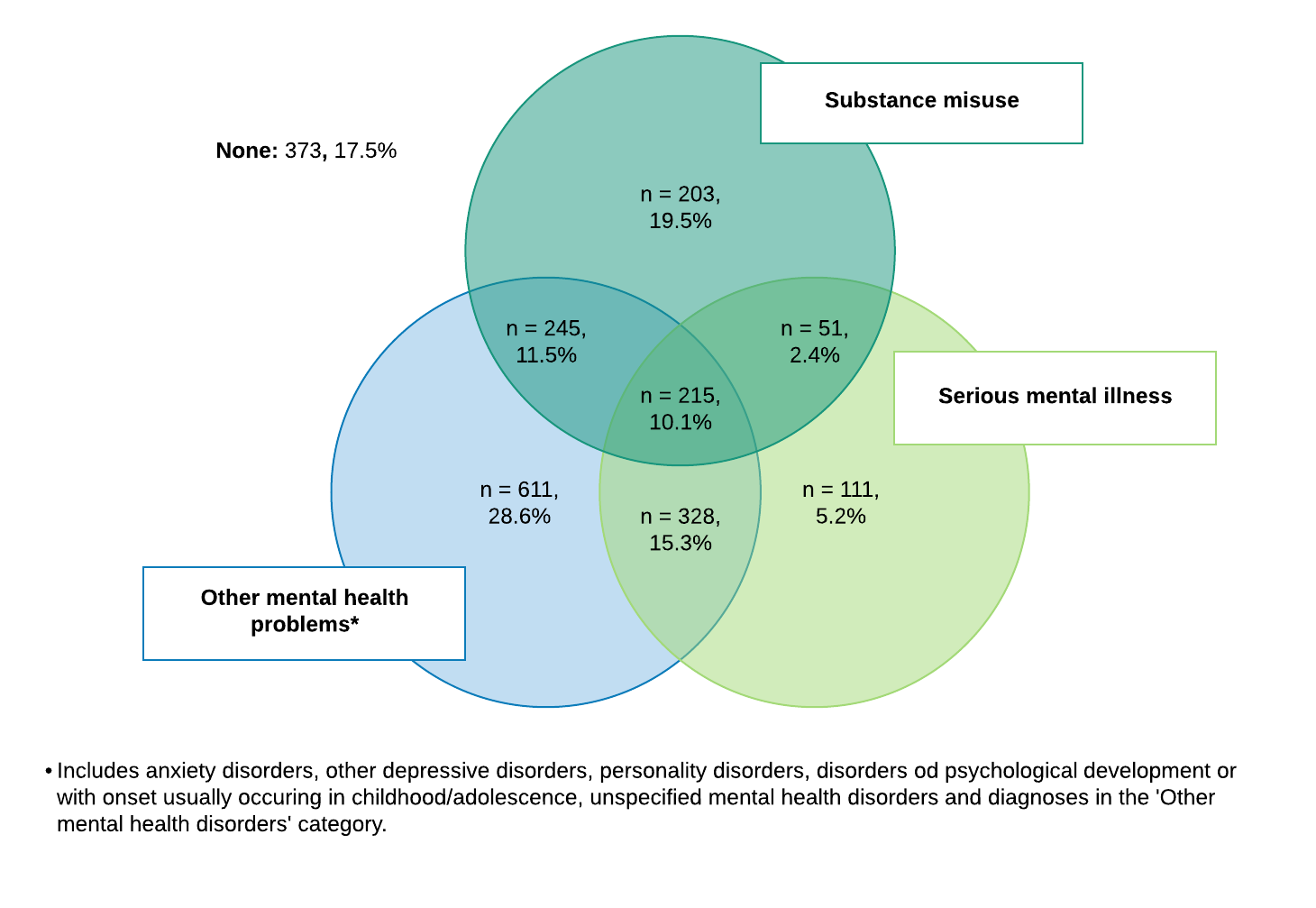


Table S 1: STROBE checklist

|  | **Item No** | **Recommendation** | **Page  No** |
| --- | --- | --- | --- |
| **Title and abstract** | 1 | (*a*) Indicate the study’s design with a commonly used term in the title or the abstract | 1 |
|  |  | (*b*) Provide in the abstract an informative and balanced summary of what was done and what was found | 2 |
| **Introduction** | | | |
| Background/rationale | 2 | Explain the scientific background and rationale for the investigation being reported | 3 |
| Objectives | 3 | State specific objectives, including any prespecified hypotheses | 3 |
| **Methods** | | | |
| Study design | 4 | Present key elements of study design early in the paper | 3-4 |
| Setting | 5 | Describe the setting, locations, and relevant dates, including periods of recruitment, exposure, follow-up, and data collection | 3-4 |
| Participants | 6 | (*a*) *Cohort study*—Give the eligibility criteria, and the sources and methods of selection of participants. Describe methods of follow-up  *Case-control study*—Give the eligibility criteria, and the sources and methods of case ascertainment and control selection. Give the rationale for the choice of cases and controls  *Cross-sectional study*—Give the eligibility criteria, and the sources and methods of selection of participants | 4 |
|  |  | (*b*) *Cohort study*—For matched studies, give matching criteria and number of exposed and unexposed  *Case-control study*—For matched studies, give matching criteria and the number of controls per case | 4 |
| Variables | 7 | Clearly define all outcomes, exposures, predictors, potential confounders, and effect modifiers. Give diagnostic criteria, if applicable | 5-6 |
| Data sources/ measurement | 8* | For each variable of interest, give sources of data and details of methods of assessment (measurement). Describe comparability of assessment methods if there is more than one group | 5-6 |
| Bias | 9 | Describe any efforts to address potential sources of bias | 4 (matching controls), 6-7 (immortal time bias) |
| Study size | 10 | Explain how the study size was arrived at | 4 |
| Quantitative variables | 11 | Explain how quantitative variables were handled in the analyses. If applicable, describe which groupings were chosen and why | 6 |
| Statistical methods | 12 | (*a*) Describe all statistical methods, including those used to control for confounding | 6-7 |
|  |  | (*b*) Describe any methods used to examine subgroups and interactions | N/A |
|  |  | (*c*) Explain how missing data were addressed | 5, 14 |
|  |  | (*d*) *Cohort study*—If applicable, explain how loss to follow-up was addressed  *Case-control study*—If applicable, explain how matching of cases and controls was addressed  *Cross-sectional study*—If applicable, describe analytical methods taking account of sampling strategy | 4 (matching methods) |
|  |  | (*e*) Describe any sensitivity analyses | 6 |

| Results | | | |
| --- | --- | --- | --- |
| Participants | 13* | (a) Report numbers of individuals at each stage of study—eg numbers potentially eligible, examined for eligibility, confirmed eligible, included in the study, completing follow-up, and analysed | 4, 6 |
|  |  | (b) Give reasons for non-participation at each stage | N/A |
|  |  | (c) Consider use of a flow diagram | Fig S2 |
| Descriptive data | 14* | (a) Give characteristics of study participants (eg demographic, clinical, social) and information on exposures and potential confounders | 4, 8-12 |
|  |  | (b) Indicate number of participants with missing data for each variable of interest | 8 |
|  |  | (c) *Cohort study*—Summarise follow-up time (eg, average and total amount) | 8 |
| Outcome data | 15* | *Cohort study*—Report numbers of outcome events or summary measures over time | 8-12 |
|  |  | *Case-control study—*Report numbers in each exposure category, or summary measures of exposure | N/A |
|  |  | *Cross-sectional study—*Report numbers of outcome events or summary measures | N/A |
| Main results | 16 | (*a*) Give unadjusted estimates and, if applicable, confounder-adjusted estimates and their precision (eg, 95% confidence interval). Make clear which confounders were adjusted for and why they were included | Table S4 |
|  |  | (*b*) Report category boundaries when continuous variables were categorized | N/A |
|  |  | (*c*) If relevant, consider translating estimates of relative risk into absolute risk for a meaningful time period | 12, Table S5 |
| Other analyses | 17 | Report other analyses done—eg analyses of subgroups and interactions, and sensitivity analyses | Table S4 |
| Discussion | | | |
| Key results | 18 | Summarise key results with reference to study objectives | 13 |
| Limitations | 19 | Discuss limitations of the study, taking into account sources of potential bias or imprecision. Discuss both direction and magnitude of any potential bias | 14 |
| Interpretation | 20 | Give a cautious overall interpretation of results considering objectives, limitations, multiplicity of analyses, results from similar studies, and other relevant evidence | 13-15 |
| Generalisability | 21 | Discuss the generalisability (external validity) of the study results | 13-14 |
| Other information | | | |
| Funding | 22 | Give the source of funding and the role of the funders for the present study and, if applicable, for the original study on which the present article is based | Funding statement |

*Give information separately for cases and controls in case-control studies and, if applicable, for exposed and unexposed groups in cohort and cross-sectional studies.

**Note:** An Explanation and Elaboration article discusses each checklist item and gives methodological background and published examples of transparent reporting. The STROBE checklist is best used in conjunction with this article (freely available on the Web sites of PLoS Medicine at http://www.plosmedicine.org/, Annals of Internal Medicine at http://www.annals.org/, and Epidemiology at http://www.epidem.com/). Information on the STROBE Initiative is available at www.strobe-statement.org.

Table S 2: Cox proportional hazards model results (top two rows – final reported model; bottom two rows – final model but without ‘Involvement in care proceedings’ as a time-dependent covariate)

| Covariate | Model with time-varying covariate? | Hazard Ratio (95% confidence Interval) | Standard error | P-value |
| --- | --- | --- | --- | --- |
| Involvement in care proceedings (0 = no, 1 = yes) | Yes | 2.15 (1.68 to 2.74) | 0.12 | < 0.001 |
| Age at first SLaM contact  (years) | - | 1.08 (1.08 to 1.09) | 0.004 | < 0.001 |
| Involvement in care proceedings (0 = no, 1 = yes) | No | 1.66 (1.30 to 2.12) | 0.12 | < 0.001 |
| Age at first SLaM contact  (years) | - | 1.08 (1.07 to 2.09) | 0.004 | < 0.001 |

Note: 3 of the matched controls had a death date preceding their first contact with SLaM and were excluded from this analysis (0.5% of matched controls who died and 0.02% of all matched controls).

Table S 3: Distribution of the matching variable, by cohort

|  | Frequency (%) or Median [25%, 75% quantile]  among women using SLaM services | |
| --- | --- | --- |
| **Matching variables** | Cases  (n = 2137) | Matched controls  (n = 17,096) |
| **Electronic patient record system*** |  |  |
| ePJS record only | 922 (43.1) | 7376 (43.1) |
| Iaptus record only | 189 ( 8.8) | 1512 ( 8.8) |
| Both | 1026 (48.0) | 8208 (48.0) |
|  |  |  |
| **Follow-up time** |  |  |
| Time from first SLaM contact to end of study (31^st^ March 2020) or death | 10.63 [7.01, 13.19] | 10.59 [6.99, 13.16] |

*ePJS is the electronic patient record system used by all SLaM services except for the four IAPT (Improving access to psychological therapies) services, which use the Iaptus electronic patient record system.

Table S 4: Age and ethnicity, by cohort

|  | Frequency (%) or Median [25%, 75% quantile]  among women using SLaM services | |
| --- | --- | --- |
|  | Cases  (n = 2137) | Matched controls  (n = 17,096) |
| **Age at first SLaM contact** |  |  |
| 0-17 years | 288 (13.5) | 2218 (13.0) |
| 18-24 | 425 (19.9) | 2906 (17.0) |
| 25-29 | 431 (20.2) | 2594 (15.2) |
| 30-34 | 364 (17.0) | 2399 (14.0) |
| 35-39 | 299 (14.0) | 2217 (13.0) |
| 40-44 | 193 ( 9.0) | 1921 (11.2) |
| 45-49 | 94 ( 4.4) | 1633 ( 9.6) |
| 50+ | 43 ( 2.0) | 1208 ( 7.1) |
|  |  |  |
| **Ethnicity** |  |  |
| White | 1044 (48.9) | 9061 (53.0) |
| Black or Black British | 701 (32.8) | 3823 (22.4) |
| Asian or Asian British | 44 ( 2.1) | 428 ( 2.5) |
| Mixed heritage | 149 ( 7.0) | 768 ( 4.5) |
| Other | 143 ( 6.7) | 1784 (10.4) |
| Unknown | 56 ( 2.6) | 1232 ( 7.2) |

Table S 5: Substance-related psychiatric diagnoses, by cohort

|  | Frequency (%) or Median [25%, 75% quantile]  among women using SLaM services | |
| --- | --- | --- |
| **Substance-related psychiatric diagnoses** | Cases  (n = 2137) | Matched controls  (n = 17,096) |
| Drug-related diagnoses | 464 (21.7) | 998 ( 5.8) |
| Alcohol-related diagnoses | 290 (13.6) | 978 ( 5.7) |

Table S 6: Estimated 5 and 10-year mortality rates for women aged 21, 28, and 36 years old at first SLaM contact

|  |  | Estimated mortality rate | |
| --- | --- | --- | --- |
| Time since first SLaM contact (years) | Age at first SLaM contact | Cases | Matched controls |
| 5 | 21 years | 0.73% (0.56 to 0.97) | 0.34% (0.28 to 0.42) |
| 5 | 28 years | 1.29% (0.99 to 1.66) | 0.60% (0.51 to 0.71) |
| 5 | 36 years | 2.43% (1.90 to 3.12) | 1.14% (0.99 to 1.31) |
| 10 | 21 years | 1.79% (1.39 to 2.30) | 0.84% (0.70 to 1.01) |
| 10 | 28 years | 3.12% (2.47 to 3.94) | 1.46% (1.27 to 1.68) |
| 10 | 36 years | 5.85% (4.67 to 7.32) | 2.77% (2.50 to 3.07) |
| *the ages 21, 28 and 36 are the 25%, 50% (median) and 75% quantiles of age at first SLaM contact among the care proceedings cohort.  Note: 3 of the matched controls had a death date preceding their first contact with SLaM and were excluded from this analysis (0.5% of matched controls who died and 0.02% of all matched controls). | | | |
